# β-Catenin Is Critical for Cerebellar Foliation and Lamination

**DOI:** 10.1371/journal.pone.0064451

**Published:** 2013-05-17

**Authors:** Jing Wen, Hong-Bin Yang, Bing Zhou, Hui-Fang Lou, Shumin Duan

**Affiliations:** 1 Institute of Neuroscience and Key Laboratory of Neuroscience, Shanghai Institutes for Biological Sciences, Chinese Academy of Sciences, Shanghai, China; 2 Department of Neurobiology, Key Laboratory of Medical Neurobiology of the Ministry of Health of China, Key Laboratory of Neurobiology of Zhejiang Province, Zhejiang University School of Medicine, Hangzhou, China; Tokyo Medical and Dental University, Japan

## Abstract

The cerebellum has a conserved foliation pattern and a well-organized layered structure. The process of foliation and lamination begins around birth. β-catenin is a downstream molecule of Wnt signaling pathway, which plays a critical role in tissue organization. Lack of β-catenin at early embryonic stages leads to either prenatal or neonatal death, therefore it has been difficult to resolve its role in cerebellar foliation and lamination. Here we used *GFAP-Cre* to ablate *β-catenin* in neuronal cells of the cerebellum after embryonic day 12.5, and found an unexpected role of β-catenin in determination of the foliation pattern. In the mutant mice, the positions of fissure formation were changed, and the meninges were improperly incorporated into fissures. At later stages, some lobules were formed by Purkinje cells remaining in deep regions of the cerebellum and the laminar structure was dramatically altered. Our results suggest that β-catenin is critical for cerebellar foliation and lamination. We also found a non cell-autonomous role of β-catenin in some developmental properties of major cerebellar cell types during specific stages.

## Introduction

In addition to its well-known role in coordinating motor processing, recent studies indicate that the cerebellum is also important for some higher order brain functions, including cognition, emotion, and language processing [1]. Morphogenesis of central nervous system (CNS) is a complex and highly-ordered developmental process and is critical for normal brain function. The relatively few well-defined cell types and the stereotypical foliation pattern make the cerebellum an ideal model system for studying CNS morphogenesis. During development it is transformed from a curved, sausage-shaped structure into one with deep fissures and large finger-like appendages called folia [2]. All mammals have a similar basic pattern of ten folia in the medial cerebellum (vermis), and these folia are stereotypical in structure between animals of the same species, suggesting that foliation may be genetically determined [3]. Folia may serve as a broad platform on which the anterior-posterior organization of the sensory-motor circuits of the cerebellum is built [4], [5]. It is thus important to understand how folia arise. It has been proposed that a key event in the initiation of foliation is the acquisition of a distinct cytoarchitecture in the regions that will become the base of each fissure. These regions are termed ‘anchoring centers’. Afterwards, folia grow by lengthening, while the bases of the fissures are largely fixed in position [3]. A few molecules have been demonstrated to affect the positions of fissure formation [3], [6], [7], [8], [9]. The role of wnt signaling in cerebellar foliation patterning has not been investigated, despite the fact that mutation of Wnt1 results in loss of the entire cerebellum, or loss of only anterior lobules [10], [11], [12].

The cerebellum also exhibits an ordered laminar organization. Defined cell layers are formed by a series of well-orchestrated cell migration events. Purkinje cells, Bergmann glia and interneurons migrate radially from the primary germinal zone toward the cerebellar surface. Granule cell precursors (GCP) first move tangentially from the rhombic lip across the surface of the developing cerebellum. These cells then form a secondary germinal zone, the external granule cell layer (EGL), which lies between the meninges and the Purkinje cell layer (PCL). Granule cells that are generated in the EGL subsequently migrate radially along Bergmann glial fibers through the PCL to form the internal granule cell layer (IGL) [2]. During development, Bergmann fibers are known to associate with migrating granule cells and the concept of glia-guided neuronal migration has been proposed [13].

β-catenin is a downstream signaling component of Wnts [14] and plays a pivotal role in cadherin-mediated cell adhesion [15]. Wnts can influence tissue organization and growth by functioning locally, in an autocrine manner or on immediately adjacent cells, or by generating a gradient across a tissue [16]. In the cerebellum, conditional knockout of *β-catenin* at early stage of embryogenesis using the *Wnt1-Cre* promoter leads to agenesis of the cerebellum [17]. Ablation of *β-catenin* at midgestation using the *nestin-Cre* promoter causes premature neural precursor cell fate commitment [18]. Such mutant mice die around birth, while cerebellar foliation and lamination, including the formation and lengthening of different lobules, migration of granule cells from the EGL to the IGL and arrangement of the PCL from multilayers to a monolayer, are largely postnatal processes. It is unclear whether β-catenin has a function in these processes.

In the present study, we used *GFAP-Cre* to ablate *β-catenin* in neural cells. The *hGFAP-Cre* promoter activates at a later time point and in a narrower distribution than the *nestin-Cre* promoter [19]. The mutant mice lived to about three weeks after birth. They did not show obvious defects in cerebellar structure before the foliation process started and therefore provided an ideal model for studying the role of β-catenin in cerebellar foliation and lamination. Unexpectedly, we found that β-catenin was required for the positioning and formation of cerebellar fissures, implying an essential role of canonical wnt signaling in cerebellar foliation. Furthermore, β-catenin was found to be required for the organization of neuronal layers. Surprisingly, selective perturbation of β-catenin in any single type of neural cell, including Purkinje cells, Bergmann glia, or a portion of granule cells, did not cause apparent defects in their distribution or gross differentiation, suggesting a non cell-autonomous role of β-catenin in these developmental properties of major cerebellar cell types.

## Materials and Methods

### Mating and Embryos

All the animal protocols were approved by the Animal Committee of Institute of Neuroscience. To generate conditional knockout mice, the *hGFAP-Cre* line [20], *hGFAP-CreER^T2^* line [21] or *L7-Cre* line [22] was crossed with the floxed *β-catenin* line [23]. F1-generation mice inheriting the *cre* gene were crossed to *β-catenin^fl/fl^* or *β-catenin^fl/wt^* mice. F2-generation mice carrying *cre* and two floxed *β-catenin* alleles were used as the mutant type. All the other littermates were used as the control type. In some cases the ROSA26 reporter allele (*R26R*) [24] was introduced into F1 and F2 mice by crossbreeding. The *R26R* line, in which the *lacZ* reporter construct is integrated into the ROSA locus, is common to detect Cre-mediated recombination. In the absence of Cre expression, the *lacZ*-encoding protein β-galactosidase (β-Gal) is not expressed due to a floxed stop signal. Cre expression results in the removal of the stop signal and expression of β-Gal, which could cleave 5-bromo-4-chloro-indolyl-β-D-galactopyranoside (Xgal) and form an intensely blue product. The results obtained from the control or mutant mice were consistent with or without the *R26R* allele, so we did not distinguish them in the descriptions below. In experiments relating to timed pregnancies, the plug date was defined as embryonic day E0.5.

### Histology and Immunohistochemistry

Mice were deeply anesthetized with Pentobarbital sodium salt (Sigma) and perfused with 0.9% NaCl, followed by 3% or 4% paraformaldehyde/Phosphate buffered saline (PBS). Brains were removed and postfixed for 2–5 h for Xgal staining, or overnight for immunohistochemical staining. After cryoprotection in 30% sucrose and subsequent freezing in Optimal Cutting Temperature compound (OCT), sections were cut 30–40 μm in thickness on a cryostat and either placed in PBS or allowed to air-dry on slides. Sections were stained with hematoxylin and eosin following the manufacturer's instructions (Beyotime Institute of Biotechnology). For Xgal histochemistry, sections were stained for 2 h to overnight at 37°C in Xgal staining solution which consisted of the staining buffer (in mM: 8.1 Na_2_HPO_4_, 1.9 NaH_2_PO_4_, 154 NaCl, 2 MgCl_2_, 5 K_3_[Fe(CN)_6_], and 5 K_4_[Fe(CN)_6_]), 0.5% Triton X-100 and 1% Xgal stock solution (100 mg/ml, Ameresco).

For immunostaining, sections or fixed cultured cells were permeabilized with 0.3% Triton X-100 in PBS for 30 min before treatment with 10% BSA for 1 h at room temperature. Sections were then stained with one or two of the following antibodies overnight at 4°C: rabbit anti-GFAP (1∶500, Chemicon, AB5804), mouse anti-GFAP (1∶50, DakoCytomation, M0761), mouse anti β-catenin (1∶1000, BD Transduction Laboratories, 610154), rabbit anti-calbindin-D28K (1∶3000, Sigma, C7354), mouse anti-NeuN (1∶100, Chemicon, MAB377), rabbit anti-BLBP (1∶1000, Abcam or Chemicon, AB9558), mouse anti-nestin (1∶1500, Chemicon, MAB353), 4D7 anti-TAG1 (1∶4, supernatant, Developmental Studies Hybridoma Bank), and rabbit anti-laminin (1∶300, Sigma, L2020). For immunohistochemistry using the rabbit anti-phospho histone H3 antibody (1∶200, Millipore, 06–570), slides were subjected to antigen retrieval by heating in a microwave at 30% power for 7 min in 10 mM sodium citrate, pH 6.0. Then the slides were left to cool at room temperature for 45 min before washing in PBS and the permeabilization step. After washing to remove excess primary antibodies, the sections were incubated for 2 h at room temperature with Alexa fluro 488, cy3, or cy5, or TRITC-conjugated secondary antibodies.

In some experiments, staining was performed using the HRP/DAB-based staining system (Zhongshan Golden Bridge Biotechnology Co., Ltd) according to the manufacturer's specifications.

Images were collected with the laser confocal microscope (Olympus IX71) driven by Fluoview 500 software, or the NIKON E600FN microscope equipped with an automatic stage driven by the Neurolucida software.

### Cell Culture and siRNA transfection

Astrocytes were prepared from the cortex and hippocampus of C57/BL6 mice on P0, and cultured in minimal essential medium (MEM) with 10% FBS (Gibco). After 7–10 days when cells reached confluence, they were detached with trypsin and transfected with plasmid DNA by nucleofection (Amaxa). Vector-based siRNA against β-catenin was encoded by pSUPER vectors, with the described sequence (5′-ACATAATGAGGACCTACAC-3′). An siRNA that does not target any known mammalian gene (5′-TTCTC CGAACGTGTCACGT-3′) was cloned into pSUPER as a control [23].

### Cerebellar *in vivo* electroporation

Cerebellar *in vivo* electroporation was conducted as described previously [25]. Briefly, P4 C57/BL6 mice were anesthetized, after which the parietal bone was exposed and pierced, and DNA containing 0.01% fast green was injected onto the surface of the cerebellar cortex. Electroporation (5 pulses of 50 ms square pulses of 90–100 V at 950 ms intervals) was then carried out using an Electro Square Porator (CUY21, NEPA GENE).

### Infection with Adenoviruses

Ad5-GFP (∼1×10^9^ pfu/ml), which expresses EGFP driven by the mCMV promoter, was purchased from Vector Gene Technology Co. Ltd; AdCreM2 (5×10^9^ pfu/ml), which expresses Cre driven by the mCMV promoter, was purchased from Microbix Biosystems Inc.

*In utero* virus injection was performed as previously described [26]. In brief, timed pregnant mice were anesthetized, a 3-cm midline laparotomy was performed, and the uterus was externalized. About 1 μl adenoviral vectors was injected into the midbrain ventricle of embryos with a pulled glass capillary. Each injected embryo was immediately laid cerebellum-down, and held for 10 sec. After most or all of the embryos were injected, the uterine horns were repositioned in the abdominal cavity, and the abdominal wall and skin were sutured.

Postnatal virus injection was also performed as described [27]. P0 mice were anesthetized with ice. Then they were put onto an ice-bag and mounted in a stereotactic frame with an adaptor. A midline incision was made, the skin over the midbrain and cerebellum was opened, and the muscle over the cranium was carefully incised. A syringe (NF33BV, World Precision Instruments) was used to rapidly penetrate and make a hole in the dura. Then it was drawn out and reinserted though the hole, and held ∼1.3 mm below the dura. After 2 min, the syringe was retracted 0.3 mm, to form a slight pocket in the parenchyma. After a pause of 2 min for pressure equalization, 0.5 μl virus-containing solution was injected at a rate of 0.5 μl per minute. Afterwards, the syringe was left in place for an additional 2 min, then withdrawn 0.3 mm, and the process repeated. A total of 4 injections were made at different depths. For co-expression of GFP and Cre, Ad5-GFP was mixed with AdCreM2 at 8∶1, and 0.01% fast green was added to the mixture.

## Results

### *hGFAP-Cre-*mediated deletion of *β-catenin* in the cerebellum

Initiation of cerebellar foliation usually starts at E17.5, when three slight indentations form at specific positions on the surface, representing the principal fissures. Later, the fissures become deeper and more fissures are formed. At the postnatal stage, granule neurons from the EGL migrate inward to assume a position beneath the PCL, forming the IGL [3]. To study the role of β-catenin in cerebellar foliation and lamination, we crossed *β-catenin^fl/fl^* mice with *hGFAP-Cre* mice.

**Figure 1:**
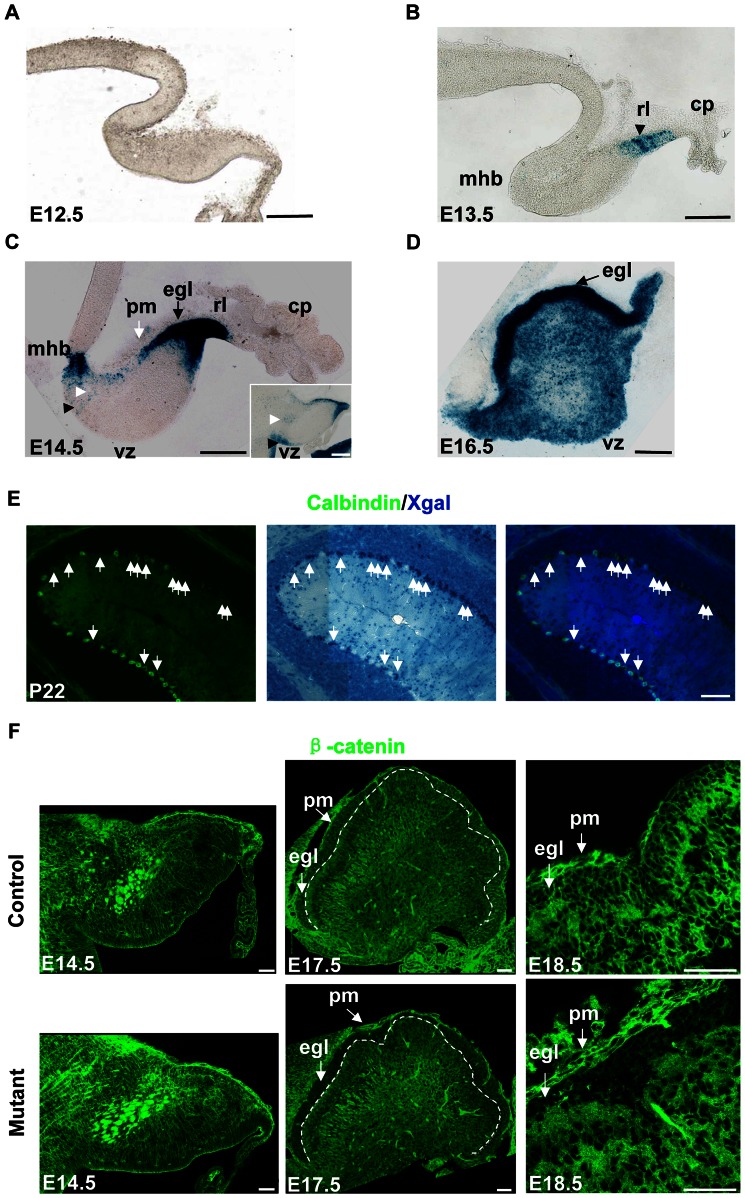
*hGFAP-Cre* activity in the cerebellum. Sagittal sections were shown. (A) Xgal staining in an E12.5 *hGFAP-Cre, R26R* mouse, showing the absence of Cre activity in the cerebellum. (B) Xgal staining of an E13.5 *hGFAP-Cre, R26R, β-catenin^fl/fl^* mouse. The rl (black arrowhead) showed Xgal labeling. (C) Xgal staining of an E14.5 *hGFAP-Cre, R26R, β-catenin^fl/wt^* mouse. The rl and egl showed extensive Xgal labeling. Xgal labeling also appeared in the mhb, vz (black arrowhead) and the interior of cerebellum (white arrowhead). The inset is a lateral section from the same mouse, showing more staining in the vz region. There is no staining in the pm (white arrow). (D) Xgal staining of an E16.5 *hGFAP-Cre, R26R, β-catenin^fl/wt^* mouse. The egl (arrow) and the interior of the cerebellum showed extensive Cre activity. (E) A P22 *hGFAP-Cre, R26R* mouse first stained with Xgal, and then stained with calbindin antibody using a fluorescent secondary antibody. Some cells were double-positive (arrows) and formed Xgal clusters. (F) Sections from E14.5, E17.5, and E18.5 *hGFAP-Cre, β-catenin^fl/fl^* mice and control littermates stained with anti-β-catenin antibody. The level of β-catenin was normal in the E14.5 mutant mouse, but clearly decreased in the EGL of E17.5 or E18.5 mutant mice. The white dashed line in E17.5 sections indicates the inner border of the EGL. β-catenin was still expressed in the pm of mutant mice. Abbreviations: cp, choroid plexus; egl, external granule cell layer; mhb, mid-hindbrain boundary; pm, pia mater; rl, rhombic lip; vz, ventricular zone. Scale bar: 100 μm (A–E) and 50 μm (F).

To determine hGFAP-Cre activity in the cerebellum, we included the *R26R* allele in the analysis. Cre-mediated recombination of the *R26R* allele results in constitutive expression of β-Gal in Cre-expressing cells and their progeny, and can be detected by Xgal. Xgal staining of embryos carrying the *hGFAP-Cre* and *R26R* alleles revealed that Cre-recombination first appeared in the rhombic lip (RL) at E13.5 (Fig. 1A and B). At E14.5, Cre recombination was found in the EGL which originates from the RL, the mid-hindbrain boundary (MHB), the ventricular zone (VZ) and the interior of the cerebellum (Fig. 1C). At E16.5, the entire dorsal surface of the cerebellum was covered by the EGL, which displayed robust Cre-mediated recombination. There were also extensive staining in the VZ and interior region (Fig. 1D). It has been reported that all the Bergmann glia and 39% of Purkinje cells express Cre recombinase in the *hGFAP-Cre* line used in the present study [20]. Indeed, some Purkinje cells stained positive for Xgal. The Xgal-positive Purkinje cells scattered throughout the cerebellum, mixing up with Xgal-negative Purkinje cells both in the *hGFAP-Cre, R26R* mouse and the *hGFAP-Cre, R26R, β-catenin^fl/fl^* mouse (Fig. 1E and Fig. S1).

We also examined β-catenin protein expression in the *hGFAP-Cre, β-catenin^fl/fl^* mice and their littermates. In the mutant mice, downregulation of β-catenin was not evident at E14.5, but was evident in the EGL at E17.5 and E18.5 (Fig. 1F). Meningeal cells did not show Cre-mediated recombination (Fig. 1C). Neither did they show β-catenin downregulation (Fig. 1F). Heterozygous *hGFAP-Cre, β-catenin^fl/wt^* mice did not show alterations in the β-catenin expression level (data not shown).

### Abnormal foliation of the cerebellum in *hGFAP-Cre, β-catenin^fl/fl^* mice

**Figure 2:**
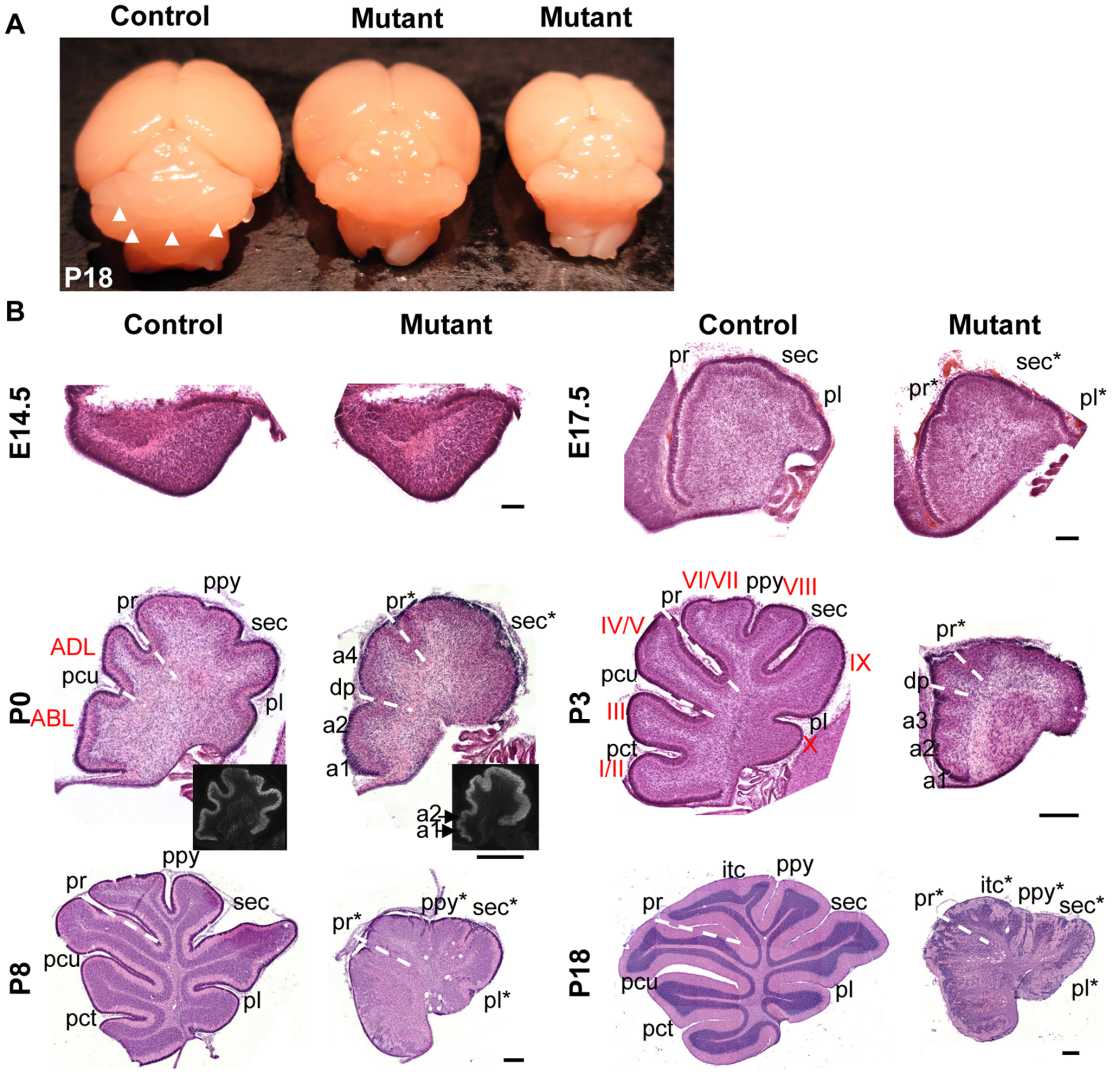
Foliation defects in the *hGFAP-Cre, β-catenin^fl/fl^* mouse. (A) The surface of a control P18 cerebellum was separated by several grooves (white arrowheads). The cerebella of its mutant littermates were smaller and had a smooth surface. (B) Midsagittal sections stained with hematoxylin and eosin. In the mutant mice recognizable fissures were designated by an asterisk after the corresponding name. Other fissures were assigned from rostral to caudal as ‘a1, a2...an’ so that the number and the sequence of these fissures are easy to be identified. At E14.5 and E17.5, the cerebella of mutant mice showed no evident difference from control mice. The pattern of fissures changed in the mutant cerebellum at P0 and P3, with more fissures in the rostral part and fewer fissures in the caudal part. The white dotted line indicates the position of the pr, recognized as the boundary between the anterior raised part and the posterior sloped part, or the deepest fissure in the anterior lobe (pcu in the control mice and dp in the mutant mice). The P0 mutant cerebellum had two ectopic fissures (a1 and a2) anterior to the dp and one ectopic fissure between the dp and pr (a4). As a result, the shapes of ABL and ADL were changed, respectively. The inset is immunostaining of calbindin, showing more clear structure of fissures a1 and a2. The P3 mutant cerebellum had three fissures anterior to the dp (a1, a2 and a3). The control cerebellum had only one (pct). At P8 and P18, the laminar structure was disordered and fissures were formed in the caudal part of the mutant cerebellum. Rostral is to the left and dorsal is to the top in each photograph. Abbreviations: ABL, anterobasal lobe; ADL, anterodorsal lobe; itc, intercrural; pct, precentral; pcu, preculminate; pl, posterolateral; ppy, prepyramidal; pr, primary; sec, secondary fissure. Scale bars: 100 μm in E14.5 and E17.5 sections, and 250 μm in P0-P18 sections.

The *hGFAP-Cre, β-catenin^fl/fl^* mice were ataxic (Movie S1) and lived for ∼3 weeks. Out of 50 mutant mice examined, only one female survived for more than one month. The mutant cerebella were smaller than those of wild-type littermates. The surface was smooth and lacked fissures (Fig. 2A). The only survivor lived for 6 months until it was killed. It was not fertile and also showed abnormal motor coordination, with a small and smooth cerebellum.

**Figure 3:**
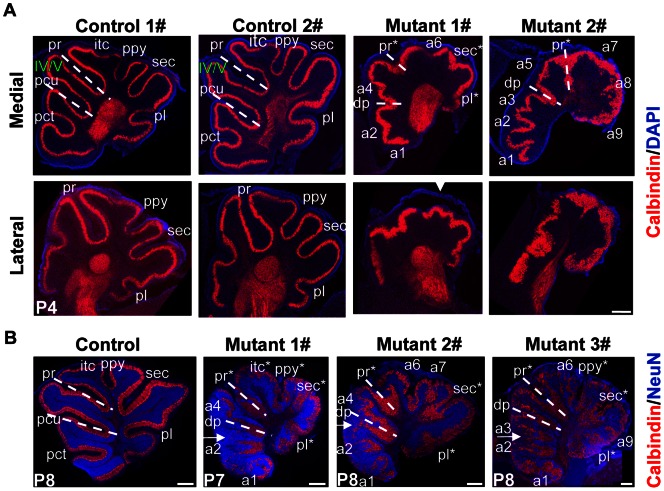
Variable foliation patterns among *hGFAP-Cre, β-catenin^fl/fl^* mice. The white dotted line indicates the position of the pr, or the deepest fissure in the anterior lobe (pcu in the control mice and dp in the mutant mice). In the mutant mice recognizable fissures were designated by an asterisk after the corresponding name. Other fissures were assigned from rostral to caudal as ‘a1, a2...an’. (A) Example medial and lateral sections of four P4 littermates stained for calbindin and counterstained with DAPI. While the foliation patterns were similar in the two control cerebella, they were different in the two mutant cerebella. In both mutant 1# and mutant 2#, one ectopic fissure appeared between the two dotted lines (a4 and a5, respectively). Therefore the shape of Lobule IV/V was changed. From anterior to deepest fissure, there is only one fissure (pct) in the control mice, but there were two or more fissures in the mutant mice (a1 and a2 in mutant 1#; a1, a2 and a3 in mutant 2#). Lateral sections showed simpler folia than medial sections. Note that the mutant egl tended to be torn off (arrowhead). (B) Example medial sections of one control and three mutant cerebella at P7 or P8 stained with antibodies to calbindin and NeuN. In mutant 1# and mutant 2# one ectopic fissure (a4) appeared between the two dotted lines. There were more fissures anterior to dp in all the three mutant cerebella (a1 and a2 in mutant 1# and 2#; a1, a2 and a3 in mutant 3#). Arrows indicate the deeply-located lobules. Rostral is to the left and dorsal is to the top in each photograph. Abbreviations: itc, intercrural; pct, precentral; pcu, preculminate; pl, posterolateral; ppy, prepyramidal; pr, primary; sec, secondary fissure. Scale bars: 250 μm.

Histological analysis of sagittal sections from *β-catenin* mutant mice demonstrated that at E14.5, the mutant cerebellum did not show discernible abnormalities in structure (Fig. 2B). At E17.5, the PCL invaginated at specific positions, and the EGL became thicker above the invagination sites in both control and mutant mice (Fig. 2B). At around P0, the cerebellar outer surface in control mice folded and formed fissures. Folia in the mutant mice were decreased in depth and fused. Interestingly, the positions of fissures were also altered in mutant mice. The position of primary fissure (pr), which is the boundary between the rostral raised part and the posterior sloped part, was detectable in the mutant cerebellum. Ectopic fissures appeared just anterior to the pr, or anterior to the deepest fissure (dp) in the anterior lobe. The shapes of anterodorsal lobe (ADL) and anterobasal lobe (ABL) were changed accordingly (Fig. 2B and Fig. S2A). At P3 or P4, the shapes of Lobule IV/V, Lobule I/II and Lobule III were also changed (Fig. 2B and Fig. S2B). Before P3 or P4, the central and caudal cerebella of mutant mice tended to have fewer fissures, as compared with control mice (Fig. 2B and Fig. S2). By P7 and P8, fissures formed in the central and caudal cerebellum of mutant mice (Fig. 2B), with even more complex foliation pattern (Fig. 3B). The control mice had a consistent foliation pattern. In contrast, the foliation pattern of individual mutant mice was variable, even if they were littermates (Fig. 3 and Fig. S2). Lateral sections showed simpler folia than medial sections (Fig. 3A). In addition, at P7 and P8 the apical surface of some rostral lobules was formed by Purkinje cells that remained deep in the cerebellum (Fig. 3B, arrows). Thus, β-catenin is involved not only in the positioning of cerebellar fissures, but also in localization of the PCL to the crown of some rostral lobules.

### Abnormal lamination of the cerebellum in *hGFAP-Cre, β-catenin^fl/fl^* mice

**Figure 4:**
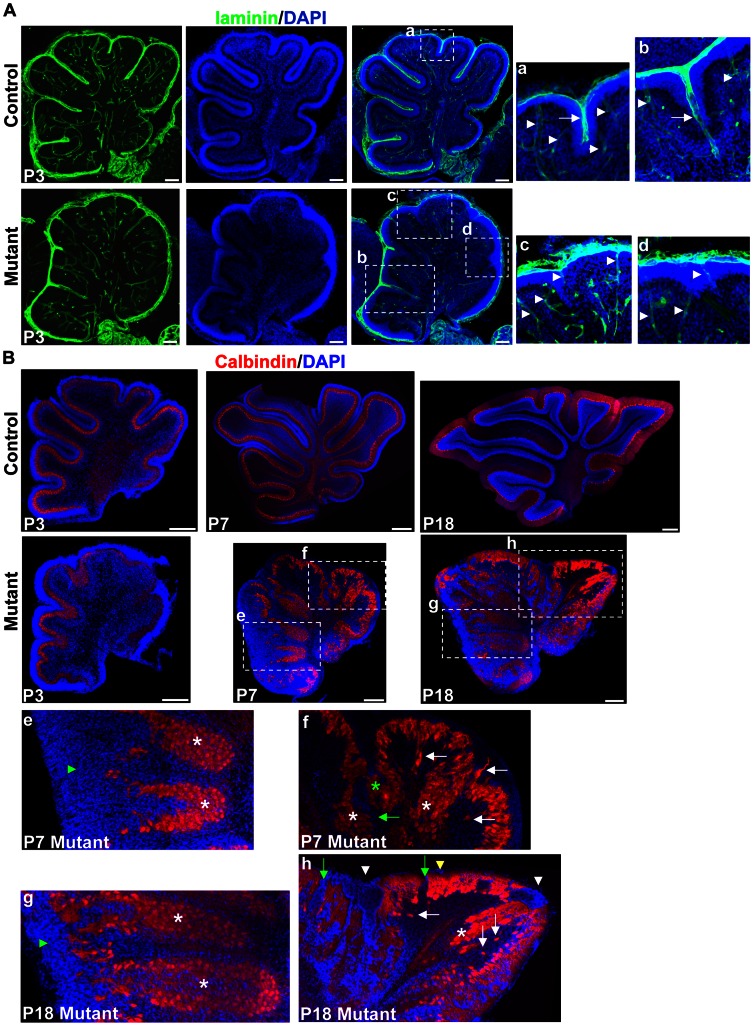
Lamination defects in the *hGFAP-Cre, β-catenin^fl/fl^* mouse. (A) Sagittal sections through the cerebellar cortex of P3 mice stained with antibody against laminin and with DAPI. The right panels are enlargements of the squares in the middle panels. Note that laminin was deposited around blood vessels in control and mutant mice (arrowheads). In control mice, laminin was incorporated into the meningeal basement membranes overlying the EGL, and penetrated into the folia at fissures (arrow in a). In mutant mice, laminin either crossed the base of granule cell accumulation (arrow in b), or did not penetrate into the granule cell accumulation (c and d). (B) Midsagittal sections stained for calbindin and DAPI. At P3, mutant cerebella had a relatively normal PCL and EGL. The PCL was disrupted at P7. The squares are enlarged in the lower panels (e, f, g and h). Green arrowheads in e and g indicate deeply-located PCL covered externally by granule cells. In f and h, white asterisks indicate fused sites of the PCL from adjacent lobules. The green asterisk indicates the PCL fused in the middle of an unsplit fissure. White arrows indicate delaminated Purkinje cells, and green arrows indicate gaps in the PCL. White arrowheads indicate granule cell ectopia at the fusion lines between adjacent lobules, and yellow arrowhead indicates granule cell ectopia below the pial surface. Scale bars: 100 μm in (A) and 250 μm in (B).

In mutant mice, adjacent lobules were fused and not properly separated by meninges (Fig. 2A and B). This was confirmed by immunostaining with antibody to laminin, a marker for meningeal basement membrane, which either crossed the base of the granule cell accumulation (Fig. 4A, b), or just covered the crown of the granule cell accumulation (Fig. 4A, c and d). This was different from control mice, in which the meningeal basement memberane covered both the base and crown of a folium (Fig. 4A, a).

Immunostaining with the Purkinje cell marker calbindin revealed that the PCL between adjacent lobules fused, at the base or in the middle of a fissure, or across a whole fissure in the mutant mice (Fig. 4B, white and green asterisks).

The mutant cerebellum had a well-organized laminar structure before P3, despite the fissures being unsplit. After P3 the laminar structure became disordered. In the P7 mutant mice, outside the apical surface of the deeply-located PCL in the rostral vermis, the granule cell layer thickened (Fig. 4B, e). Many gaps formed in the PCL, and some Purkinje cells delaminated from the PCL (Fig. 4B, f, green and white arrows). At P18 in control mice, granule cells were all in the IGL. In mutant mice ectopic granule cells were found outside deeply-located PCL (Fig. 4B, g), along the fusion lines between folia and below the pial surface (Fig. 4B, h, arrowheads). Therefore, β-catenin is required for the maintenance of a well-organized layered structure in the cerebellum, which would be caused by its effects on migration or stratification of granule cells and Purkinje cells.

### Defects of granule cells in *hGFAP-Cre, β-catenin^fl/fl^* mice

**Figure 5:**
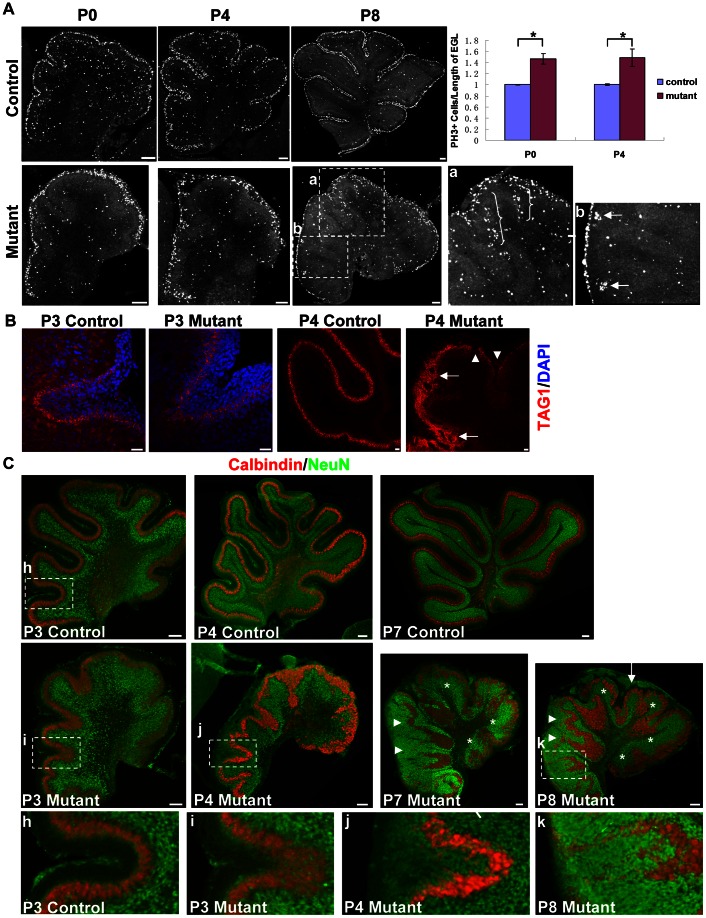
Proliferation and differentiation of granule cells in the *hGFAP-Cre, β-catenin^fl/fl^* mouse. (A) PH3 immunostaining showed that the proliferation zone in the EGL was largely maintained with no apparent ectopic proliferation at P0 and P4. At P8, some ectopic cell accumulations formed, more clearly seen in the enlargement of square a (brackets). PH3-positive cells formed small clusters at anterior fissures (arrows in b). The right panel is quantification of PH3-expressing cells in the EGL. Counted cells were divided by the length of the EGL (measured along the outer surface). The results were normalized to the mean of control cerebella and presented as mean ± SE. 3–5 slices from 2 or 3 mice were used for each group. Because the caudal part of the EGL in some P4 mutant mice tended to be torn off, these regions were excluded from the assay. Differences were statistically significant (p<0.05) as determined by Mann-Whitney two-sample rank sum test. (B) Immunohistochemistry for TAG1. At P3 in the inner EGL of both control and mutant cerebellum, an intact TAG1-expressing layer was seen, which became discontinuous and disordered in the P4 mutant cerebellum (arrowheads and arrows). (C) Immunohistochemistry for NeuN. Sections were also stained with anti-calbindin antibody and DAPI. In the control cerebellum, NeuN was expressed in the inner EGL and IGL. At P3, the mutant cerebellum still had this pattern. Later, ectopic NeuN was expressed by granule cells outside the deeply-located PCL (arrowheads), or by granule cells in an unseparated fissure (enlargements of squares j and k, compared with enlargement of square h in the bottom panel). P7 and P8 mutant cerebella are shown; NeuN+ granule cell ectopia were seen adjoining the pial surface of the P8 mutant cerebellum (arrow), but were rarely seen in the P7 mutant cerebellum. Despite these defects, the IGL did exist where a relatively normal monolayer of PCL was formed (white asterisks). Scale bars: 100 μm in (A), 20 μm in (B), and 100 μm in (C).

Because the *hGFAP* promoter was mainly active in GCPs and glial cells [20], we examined the development of these cell types. Immunostaining with phospho histone H3 (PH3), a marker for mitosis, revealed that at P0 and P4, β-catenin-null GCPs were not proliferating ectopically in places other than the EGL (Fig. 5A). The proliferation rates in the EGL of mutant cerebella, represented as PH3-positive cells per length of the EGL, were significantly higher than those of the control mice (p<0.05, Fig. 5A). This might be caused by a reduced surface circumference. In other words, granule cells were not able to spread out when the surface shrank. Indeed, the thickness of the mutant EGL was significantly higher than that of the control EGL (p<0.05, data not shown), and PH3-positive cells per area of the EGL did not show significant difference (p>0.5, data not shown). As the laminar structure of the PCL became disordered, some ectopic cell accumulations formed beneath the EGL (Fig. 5A, a). PH3-positive cells still formed small clusters at anterior fissures (Fig. 5A, b). TAG1 is an early granule cell differentiation marker [28]. At P3, mutant mice showed intact TAG1 expression. At P4, the TAG1-expressing layer became discontinuous in some places and was disordered at lobule boundaries (Fig. 5B).

We also stained with antibody against NeuN, a marker for post-mitotic, differentiated granule cells [29]. In control cerebellum, NeuN was expressed in the inner EGL and IGL. At P3, the mutant cerebellum still had this expression pattern. At P7, in the mutant mice where Purkinje cells formed a monolayer near the pial surface, the IGL largely formed and NeuN was expressed (Fig. 5C, asterisks). However, some granule cells showed retention in the disrupted molecular layer formed by Purkinje cell dendrites (Fig. S3A). Above the apical surface of the deeply-located PCL in the rostral vermis, granule cells expressed NeuN (Fig. 5C, arrowheads). In unseparated fissures, the granule cells gradually expressed NeuN (Fig. 5C, i, j and k). These changes were likely caused by aberrantly migrating granule cells in an abnormal environment (Purkinje cells too deep or too thick). In these cases the differentiation zone of granule cells was still close to the PCL and away from the pial surface. However, at P7 NeuN+ cell accumulations were also found in places adjoining the pial surface in some mutant cerebella (Fig. 5C, arrow). Because the zone near the pial surface is for GCP proliferation [30], the appearance of NeuN in these places implies premature differentiation of granule cells. At P18, most, if not all, granule cells expressed NeuN, indicating that these cells had been differentiated into mature neurons (Fig. S3B).

### Defects of Bergmann glia in *hGFAP-Cre, β-catenin^fl/fl^* mice

**Figure 6:**
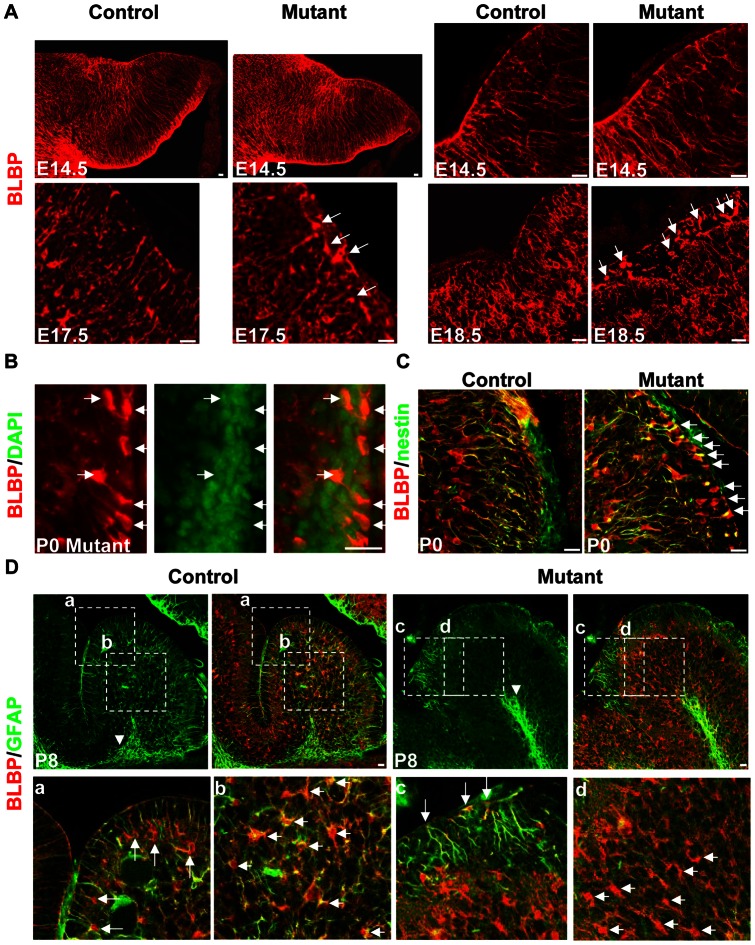
Developmental stage-dependent glial defects in the *hGFAP-Cre, β-catenin^fl/fl^* mouse. (A) Sagittal sections immunolabeled with anti-BLBP antibody. At E14.5, radial glial fibers were attached to the pial surface in both control and mutant mice. The right panels were acquired under high power (60×). At E17.5, in the caudal part of the mutant cerebellum, the density of radial fibers decreased, and some BLBP-positive cell bodies appeared in the EGL (arrows). At E18.5, more ectopic glial cells were seen in the EGL (arrows). (B) Section of a P0 mutant cerebellum stained with anti-BLBP antibody and DAPI. Colocalization indicates they were indeed cell bodies (arrows). (C) Sections from P0 control and mutant mice stained with antibodies against BLBP and nestin. Many double-positive cells were found in the mutant EGL (arrows), with a polarized appearance. (D) Sections of P8 control or mutant cerebellum double-stained with anti-BLBP and anti-GFAP antibodies. In the control mouse, GFAP was expressed in the white matter glia (arrowhead in the upper panel), Bergmann glia (arrows in a) and glia in-between (short arrows in b). In the mutant mouse, GFAP was only expressed by white matter glia (arrowhead in the upper panel) and ectopic glia in the EGL (arrows in c), but not by glial cells in-between (short arrows in d). The lower panels are enlargements of the squares in the upper panels (a–d). Scale bars: 20 μm.

Next we visualized glial cells in cerebellar sections by immunohistochemistry. We stained cerebellar sections with antibody against Brain lipid-binding protein (BLBP), which could label radial glia and astroglia. At E14.5, radial glial fibers spanned the cerebellar cortex in both control and mutant mice, with no apparent difference in alignment and density (Fig. 6A). At E17.5 in the rostral part of the mutant cerebellum, radial fibers still spanned the EGL (Fig. S4A). However, in the caudal part radial fibers decreased, and some BLBP-positive cell bodies appeared in the EGL (Fig. 6A, arrows). At later stages in both the rostral and caudal parts of the mutant cerebellum, more ectopic BLBP-positive cell bodies accumulated in the EGL (Figure S4B and Fig. 6A, arrows), with a distinguishable cell nucleus (Fig. 6B). These cells also stained positive for nestin and GFAP. They tended to extend fibers into the interior of the cerebellum, showing a polarity that was the reverse of Bergmann glia (Fig. 6C and Fig. 6D, c).

The astroglia in the interior of the mutant cerebellum expressed BLBP and was rather compact, compared with the scattered distribution of astroglia in the EGL. They had foliation pattern similar to Purkinje cells (Fig. S4C). The border was corresponding to the dendritic terminal of Purkinje cells. The processes of astroglia did exist but were not regularly aligned (Fig. S4D). They might partially support the inward migration of granule cells (Fig. S3A).

In control cerebellum, white-matter glia (Fig. 6D, arrowhead) and Bergmann glia (Fig. 6D, a) highly expressed GFAP postnatally. Glia between these two zones also expressed GFAP (Fig. 6D, b). In mutant cerebellum, GFAP was highly expressed by white-matter glia (Fig. 6D, arrowhead) and ectopic glia in the EGL (Fig. 6D, c). Glia in the intermediate zones showed decreased GFAP expression (Fig. 6D, d). Thus the distribution, morphology and differentiation of glial cells were all affected in the *hGFAP-Cre, β-catenin^fl/fl^* mice.

### Non cell-autonomous role of β-catenin in specific developmental properties of major cerebellar cell types

**Figure 7:**
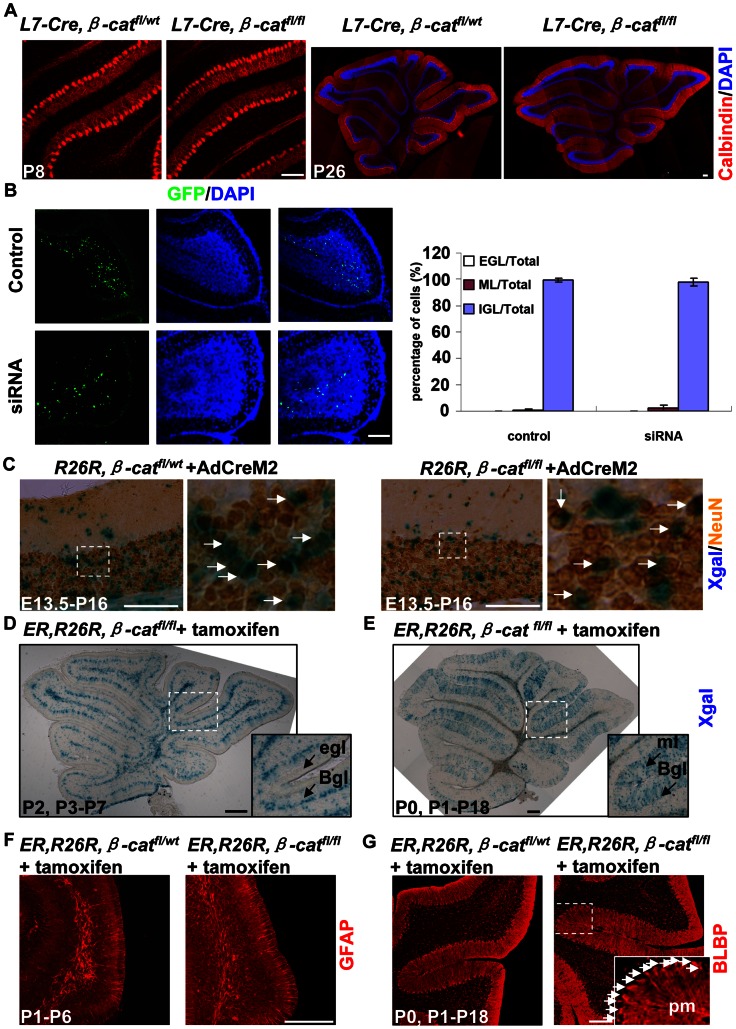
Non cell-autonomous role of β-catenin in major cerebellar cell types. (A) Sections from P8 and P26 *L7-Cre, β-catenin^fl/wt^* or *L7-Cre, β-catenin^fl/fl^* cerebellum stained with anti-calbindin antibody and DAPI. Purkinje cells from the *L7-Cre, β-catenin^fl/fl^* mouse had normal morphology and well-organized lamination, just like those from control cerebellum. (B) Wild-type C57/BL6 mice electroporated with pSuper vectors carrying either control siRNA or β-catenin siRNA at P4 and analyzed at P14. Most siRNA-transfected cells were able to migrate into the IGL. There was no significant difference between the two groups. (C) E13.5 *R26R, β-catenin^fl/wt^* or *R26R, β-catenin^fl/fl^* mice injected with AdCreM2. The mice were annalyzed at P16. Sections were first stained with Xgal and then with anti-NeuN antibody using the DAB method. Most of NeuN-positive cells were located in the IGL (arrows), indicating that Cre-expressing granule cells had migrated into the IGL. (D and E) *GFAP-CreER^T2^, R26R, β-cat ^fl/fl^* mice were induced twice between P0 and P3, and analyzed at P7 or P18, as indicated. Xgal staining showed extensive recombination in the BGL. The ml also had scattered Xgal+ cells, probably interneurons. The EGL had no Xgal+ cells. The insets are enlargements of the squares. (F and G) *GFAPCreER^T2^, R26R, β-cat^fl/wt^* mice or *GFAPCreER^T2^, R26R, β-cat^fl/fl^* mice were induced and analyzed on postnatal days as indicated, and immunostained with anti-GFAP (F) or anti-BLBP (G) antibody. In mutant cerebella, Bergmann glia also expressed BLBP and GFAP and extended fibers to the pial surface. Inset in G is the enlargement of the squared area showing glial cell bodies were seen in the BGL (arrows) and rarely seen in the ml. The slices in (E) and (G) were from the same mouse. Abbreviations: Bgl, Bergmann glia layer; egl, external granule cell layer; ml, molecular layer; pm, pia mater. Scale bars: 100 μm.

Our results showed that, in the *hGFAP-Cre, β-catenin^fl/fl^* mice, there were defects in the arrangement of Purkinje cells after P3 (Fig. 4B). To determine whether β-catenin plays a cell-autonomous role in Purkinje cell migration, we used an *L7-Cre* line to specifically ablate *β-catenin* from Purkinje cells. Cre activity is detectable as early as E17.5 [22]. We did detect Cre activity in Purkinje cells in the *L7-Cre, R26R* mice (data not shown). The *L7-Cre, β-catenin^fl/fl^* mice lived to adulthood and showed no apparent motor defects. They had inflammation in the cornea and tissues near the eyes. Inflammation was not found in *L7-Cre, β-catenin^fl/wt^* mice (data not shown). By immunostaining with anti-calbindin antibody and DAPI, we found that the morphology and lamination of Purkinje cells were both normal in the *L7-Cre, β-catenin^fl/fl^* mice (Fig. 7A). Although β-catenin may be required for Purkinje cell migration at earlier embryonic stage, our results suggest that the lamination defects appeared in the *hGFAP-Cre, β-catenin^fl/fl^* mice after P3 were unlikely caused by loss of β-catenin in Purkinje cells alone.

In the *hGFAP-Cre, β-catenin^fl/fl^* mice, Cre activity was extensively detected in GCP cells. We used two methods to assess the function of β-catenin in granule cells. First, we used an *in vivo* electroporation method. To downregulate β-catenin expression, we made an RNAi construct against β-catenin and tested its effectiveness in cultured astrocytes. Immunostaining analysis showed that β-catenin expression was significantly downregulated after transfection (Fig. S5A). We then injected this RNAi construct or its control construct beneath the cerebellar meninges and performed electroporation at P4. Most of transfected cells were GCPs as described [25]. We found that at P14 most of the GCPs transfected with β-catenin RNAi migrated to the IGL, similar to those transfected with control construct (Fig. 7B). Another method was to infect the E13.5 *R26R*, *β-catenin^fl/fl^* cerebellum with an adenovirus that was engineered to express Cre protein (AdCreM2). First we examined the effect of AdCreM2 in cultured astrocytes. The astrocytes from *β-cat^fl/fl^* mouse were first transfected with a plasmid containing a floxp-flanked stop sequence between a ubiquitous promoter and the EGFP gene. In these cells AdCreM2 efficiently excised the floxp-flanked stop sequence and initiate EGFP expression. The expression of β-catenin was apparently downregulated (Fig. S5B). Then we coinfected the *β-cat^fl/wt^* or *β-cat^fl/fl^* cerebellum with AdCreM2 and another adenovirus carrying a GFP gene (AdGFP) at E13.5. Many choroid plexus cells were infected by both viruses. In the *β-cat^fl/fl^* cerebellum these cells showed clear down regulation of β-catenin at P0 (Fig. S5C). Although the cerebellum had few GFP-positive cells (data not shown), we found that the EGL of *β-cat^fl/fl^* mouse displayed apparent down regulation of β-catenin at P0 (Fig. S5D), suggesting higher infection efficiency of AdCreM2 than AdGFP. However, Cre-mediated *β-catenin* deletion in the *R26R, β-catenin^fl/fl^* mice, as indicated by Xgal-positive staining, did not block the translocation of granule cells from the EGL to the IGL (Fig. S6A, B and Fig. 7C).

Next we determined whether the defects in Bergmann glia were the result of a cell-intrinsic effect of β-catenin loss. First, we crossed *R26R, β-catenin^fl/fl^* mice with mice bearing the *hGFAP-CreER^T2^* transgene, which expressed Cre in response to tamoxifen, to obtain *hGFAP-CreER^T2^, R26R, β-catenin^fl/fl^* mutants. Tamoxifen was given twice between P0 and P3. Xgal staining revealed that Cre-mediated recombination occurred mainly in the Bergmann glia layer (BGL), and rarely in the EGL (Fig. 7D). Some cells in other layers were also Xgal-positive; they were probably interneurons or astroglia (Fig. 7E). We induced 9 litters of mice, and 32 individuals were of the *hGFAP-CreER^T2^, R26R, β-catenin^fl/fl^* genotype. These mice showed normal cerebellar foliation and lamination (Fig. 7D and 7E). The morphology and differentiation of glial cells were also normal. There were no BLBP-positive glial cell bodies in the molecular layer (Fig. 7F and 7G). The second method was to infect the cerebellum of *R26R, β-catenin^fl/fl^* mice with AdCreM2. Injection of AdCreM2 at E14.5 affected many cells, including some Bergmann glia, whose fibers were still radially arranged and extended to the meninges, as revealed by staining with Xgal (Fig. S6C), or double-staining with Xgal and anti-BLBP antibody (Fig. S6D). Furthermore, when injection was performed with an AdCreM2 and AdGFP mixture at P0, some cells located in the BGL expressed both Cre and GFP. They were likely Bergmann glia. Their fibers were also radially arranged and extended to the meninges (Fig. S6E). This did not resemble the glial phenotype in the *hGFAP-Cre, β-catenin^fl/fl^* mice (Fig. 6). These results indicated that β-catenin did not play a cell-autonomous role in the distribution of Bergmann glia.

In summary, at the specific developmental stages we examined, β-catenin did not play a cell-autonomous role in the lamination or general differentiation of Purkinje cells (after E17.5), granule cells (after E13.5 or P4) or Bergmann glia (after E14.5 or P0).

## Discussion

We inactivated the β-catenin gene selectively in precursors of neurons and glia by Cre/Lox-mediated gene inactivation and found that the mutant mice developed severe cerebellar abnormalities. The defects included changes in the pattern of foliation, fusion of adjacent lobules, and abnormal laminar organization. The differentiation and distribution of granule cells and astroglia were also dramatically changed. However, at the stages we examined, β-catenin did not play a cell-autonomous role in the lamination or the general differentiation of major cerebellar cell types.

### β-catenin regulates the positions where fissures form

In this study, we focused on the role of β-catenin in late embryonic and early postnatal cerebellar development. Our data provide evidence that Purkinje cells formed folia-like structures with unseparated fissures, but these fissures appeared at positions different from those of wild-type mice, and different patterns formed in different mutant mice.

The proliferative kinetics of granule cells is an important factor for cerebellar foliation [31]. For example, hyperthyroidism [32], mutations in the sonic hedgehog (Shh) signaling pathway [6], loss of cilia proteins [7], or loss of cyclin D1/D2 [8], result in reduced GCP proliferation and this leads to foliation patterns that resemble an early developmental stage when the overall pattern is simpler. In contrast, hypothyroidism or overexpression of Shh leads to increased GCP proliferation and a greater number of fissures [32], [6]. Change in GCP proliferation may be associated with change in the foliation pattern of the *hGFAP-Cre, β-catenin^fl/fl^* mouse. However, the positions of ectopic fissures in the *hGFAP-Cre, β-catenin^fl/fl^* mouse was different from those in the mutant mice mentioned above [32], [6], [7], [8]. Thus factors other than GCP proliferation may also exist to affect foliation patterns.

Several molecules have been found to function in the patterning of cerebellar folia. Removal of the transcription factor Gbx2 after E9 results in lobules reduced in size to varying degrees in the vermis [33]. Mutations in the Fgf8 signaling pathway preferentially affect the growth of the anterior vermis [34], [35]. Progressive reduction of engrail1/engrail2 function moves cerebellar morphology towards a homogeneous foliation pattern throughout the medial-lateral axis [9]. Loss of engrailed-2 causes the lengths of two fissures to be reversed, but their positions are unchanged [3]. The foliation pattern of *hGFAP-Cre, β-catenin^fl/fl^* cerebella did not resemble any of these reported mutants. In addition, after P3 in the rostral part of the mutant cerebellum, some lobules remained in deep zones; while in the caudal part, lobules were still near the meninges. Some genes are differentially distributed in rostral and caudal parts of the cerebellum [36], [4], which also show differential responses to the mutation of some genes [22], [37]. The positioning of Purkinje cells in the rostral cerebellum may be more affected by the β-catenin pathway.

β-catenin is a critical molecule in the canonical Wnt signaling pathway. It acts downstream of Wnt, which is a morphogen regulating tissue patterning [15]. Our results suggest that canonical Wnt signaling regulates cerebellar folia patterning in a way different from other known molecules. Previous studies found that direct mutation of Wnt1 results in loss of the entire cerebellum, or loss of only anterior lobules [10], [11], [12], a phenotype different from that of *hGFAP-Cre, β-catenin^fl/fl^* cerebella found in the present study. It is possible that in addition to Wnt1, other members of the wnt family may be involved in mice with β-catenin mutation. Alternatively, as a partner of cadherin [15], β-catenin may affect cerebellar foliation through cell adhesion.

### β-catenin regulates the distribution of meningeal basement membrane at cerebellar fissures

Interestingly, although β-catenin was only inactivated in neurons and glia in *hGFAP-Cre, β-catenin^fl/fl^* mice, the deposition of meningeal basement membrane at cerebellar fissures was defective. The morphology of Bergmann glia has been proposed to be critical for the remodeling of meninges and the formation of fissures [3], [38], [39]. In *hGFAP-Cre, β-catenin^fl/fl^* mice, radial fibers attaching to the meninges were decreased from E17.5, and replaced by ectopic cell bodies in the EGL. This may account for the defects in the deposition of meninges. However, we cannot exclude the involvement of granule cells, which might draw the meninges into fissures together with Bergmann glia, or secrete factors to attract meningeal components into fissures. Our results also indicate that when only part of the granule cells or glial cells were depleted of β-catenin, the change in adhesive force or concentration of secreted factors was not enough to generate obvious defects in meningeal deposition.

### Non cell-autonomous role of β-catenin in lamination and general differentiation of major cerebellar cell types

β-catenin has been reported to play a cell-autonomous role in the neuronal proliferation and differentiation of cerebral cortical precursor cells [40], [41]. Activation of β-catenin in GCPs impairs proliferation and results in premature differentiation [42], [43]. Loss of β-catenin in the cerebellum at an early stage in the *Nestin-Cre, β-catenin^fl/fl^* mice leads to premature neural precursor cell fate commitment in the ventricular zone. The EGL was also disrupted at E14.5 [18]. In our study, the EGL of the *hGFAP-Cre, β-catenin^fl/fl^* mice was maintained with normal morphology until P3. It is likely that β-catenin was important in formation of the EGL from the RL, which initiates at about E13 [2], but could not be detected in our mutant mice due to the delay of β-catenin down regulation compared to Xgal expression. At around P7 in the *hGFAP-Cre, β-catenin^fl/fl^* mice, the IGL formed where a relatively normal PCL formed near the pial surface, suggesting that β-catenin-deficient granule cells were still able to migrate. Accumulation of differentiated granule cells was found beneath the meninges in some *hGFAP-Cre, β-catenin^fl/fl^* mice, suggesting premature differentiation. Our loss of function studies, by electroporating wild-type granule cells with β-catenin siRNA or infecting *R26R, β-catenin^fl/fl^* granule cells with Cre-expressing adenovirus, demonstrated a non-cell-autonomous role of β-catenin in granule cell differentiation and distribution. Several factors may contribute to the retention of granule cells in ectopic places of the *hGFAP-Cre, β-catenin^fl/fl^* mice. The gross changes of the cerebellar structure (e.g. Purkinje cells distributed too thick or too deep, or glia scaffold was destroyed) may be unpermissive for granule cell migration. Alternatively, disordered interactions of granule cells with neighboring β-catenin-deficient cells, either by adhesive force or secreted factors, may not allow granule cells to migrate.

Purkinje cells are produced in VZ at E11-13, with a peak production at E11/12. They migrated radially from the VZ towards the cortical surface between E13 and E17. Glial cells also originate from VZ. Their cell bodies migrate between E15 and P7 to form a compacted BGL. It was suggested that the arrangement of radial glia and expression of adhesion molecules may be involved in the control and guidance of Purkinje cell migration [44]. The *hGFAP* promoter was active in some VZ and cortical cells at E14.5 and E16.5, possibly including pre-migrating or migrating Purkinje cell and glial cells. However, apart from the fissures, Purkinje cells of the *hGFAP-Cre, β-catenin^fl/fl^* mice had a well-organized laminar structure until P3. This may be due to the delay of β-catenin down regulation compared to Xgal expression, or because β-catenin did not play a role in Purkinje cell migration at this stage. After P3, the laminar structure of Purkinje cells was gradually disordered. The PCL was not tightly arranged, but rather interrupted by gaps. Some Purkinje cells delaminated from the PCL. Such defects were not seen in the *L7-Cre, β-catenin^fl/fl^* mice, which expressed Cre after E17.5. The defects may be caused by changed interaction with other β-catenin-deficient cells (e.g. changed adhesive force or secreted factors), or by the disrupted glial scaffold, which became defective at as early as E17.5. The defect in astroglia at either prenatal or postnatal stage may be caused by synergistic interactions between astroglia and other cells, rather than cell-autonomous effects, as demonstrated by infection of AdCreM2 at E14.5 and induction of the *hGFAP-CreER^T2^, R26R, β-catenin^fl/fl^* mice at P0.

## Supporting Information

Movie S1 **Ataxic movement of the** ***hGFAP-Cre, β-catenin^fl/fl^*** **mouse.** A P18 mutant mouse and its control littermate are shown. The mutant mouse was unable to keep balance during walking.
(AVI)

Figure S1 **Recombination in Purkinje cells driven by the** ***hGFAP-Cre*** **promoter.** (A) Example cerebella slice stained with Xgal from a P22 *hGFAP-Cre, R26R* mouse. It was the same slice shown in Fig. 1E, which was also stained with anti-Calbindin using a fluorescent secondary antibody. Only Xgal staining was shown for clarity. Right panels are enlargement of corresponding squares labeled with a-c in the left panel. (B and C) Example cerebella slices from a P7 (B) or P16 (C) *hGFAP-Cre, R26R, β-catenin^fl/fl^* mouse stained with Xgal and anti-Calbindin antibody (DAB staining). The right panels are enlargement of corresponding squares labeled with d-h in the left panels. In all the figures Xgal-positive Purkinje cells tended to form clusters (red arrows). Such clusters scattered throughout the cerebellum and mixed up with Xgal-negative Purkinje cells (white arrows). Scale bars: 250 μm.
(TIF)

Figure S2 **Variable foliation patterns of the** ***hGFAP-Cre, β-catenin^fl/fl^*** **mice.** Midsagittal sections were analyzed by the hematoxylin and eosin method. The white dotted line indicates the position of the pr, or the deepest fissure in the anterior lobe (pcu in the control mice and dp in the mutant mice). In the mutant mice recognizable fissures were designated by an asterisk after the corresponding name. Other fissures were assigned from rostral to caudal as ‘a1, a2...an’. (A) Example sections of three E18.5 mutant mice. Mutant 1# had two fissures (a1 and a2) anterior to the dp. In the control mouse there was only one fissure (pct) anterior to the deepest fissure (pcu). Mutant 2# and 3# had one ectopic fissure between the dp and pr. The right panels are enlargement of the squared areas in the left panels (a and b). (B) Sections of six P3 or P4 mutant mice. They were different from each other. Anterior to the dp, there were two (mutant 2# and 5#) or three (mutant 1#, 3# and 6#) fissures. In the control mouse there was only one (pct). Between the pr and dp, mutant 1#, 2# and 3# had no ectopic fissure, whereas mutant 4#, 5# and 6# had one ectopic fissure (a3 in 4#; a4 in 5#; a5 in 6#). Scale bars: 100 μm.
(TIF)

Figure S3 **Distribution and differentiation of granule cells in the** ***hGFAP-Cre, β-catenin^fl/fl^*** **mouse.** (A) Parts of the P7 control and mutant cerebellum in Fig. 5 were enlarged and shown. In the mutant cerebellum many granule cells had migrated inward and formed the IGL (asterisk), whereas some granule cells showed retention in the gaps of Purkinje cell dendrite layer (arrows). (B) The P18 mutant cerebellum was stained with DAPI and antibodies against Calbindin and NeuN. Granule cells tended to accumulate and thus were largely recognized as DAPI-positive accumulations. Note that most cells in DAPI-positive accumulations were NeuN-positive (red arrows). Only very few cell accumulations were NeuN-negative (white arrows), suggesting that most granule cells had undergone differentiation. Scale bars: 100 μm in (A) and 250 μm in (B).
(TIF)

Figure S4 **Defects in Bergmann glia of the** ***hGFAP-Cre, β-catenin^fl/fl^*** **mouse.** (A) Example sections showing radial fibers spanned the EGL in the rostral part of E17.5 control and mutant cerebellum. (B) Example sections showing ectopic cell bodies appeared in the EGL of the E18.5 mutant cerebellum. (C) Neighbouring sections from a P4 mutant mouse were stained with antibodies against Calbindin or BLBP, revealing similar foliation pattern. White arrows indicate separated fissures. Yellow arrows indicate fused fissures. The green arrow indicates a fissure fused at the base. (D) Midsagittal sections from a P8 mutant mouse were stained with antibodies against Calbindin or BLBP and counterstained with DAPI. The squared region in the left panel were enlarged and shown in the three right panels. The dotted lines indicate the border of inner egl, which was corresponding to the border of dendritic terminal of Purkinje cells (a) or compact astroglia in the interior of cerebellar cortex (b). The arrowheads indicate the scattered astroglia beneath the pial surface or in the egl. Scale bars: 20 μm in (A), 100 μm in (B), (C) and (D).
(TIF)

Figure S5 **Inhibition of β-catenin expression by siRNA or Cre-expressing virus.** (A) Cultured cortical astrocytes were electroporated with pSuper vectors carrying either control siRNA or β-catenin siRNA. After 3 days, cells were fixed and immunostained with anti-β-catenin antibody. The expression level (mean fluorescence density) of β-catenin within individual GFP-positive cells was quantified. The percentages of cells within each range of expression are shown in the histograms. Differences were statistically significant (p<0.001) as determined by the Mann-Whitney two-sample rank sum test. (B) Astrocytes from *β-cat^fl/fl^* mouse were cultured and transfected with a plasmid containing a floxp-flanked stop sequence between a ubiquitous promoter and the EGFP gene (*^fl^stop^fl^-GFP*). The cells were fixed and immunostained with anti-β-catenin antibody 6 days after infection with or without AdCreM2. GFP expression was initiated and β-catenin expression was inhibited in the AdCreM2-infected cells. Note that AdCreM2 affected more cells than the *^fl^stop^fl^-GFP* plasmid, as judged by the down-regulated β-catenin expression in some GFP-negative cells. (C) E13.5 *β-catenin^fl/wt^* (upper panels) or *β-catenin^fl/fl^* (lower panels) mice injected with a viral mixture of AdCreM2 plus Ad5-GFP. The mice were killed and the choroid plexus was stained with anti-β-catenin antibody at P0. GFP-positive cells of the *β-catenin^fl/fl^* mouse showed an apparent decrease in the expression level of β-catenin (arrows). (D) The cerebella from the same mice as in (C) were stained with anti-β-catenin antibody. Note an apparent down-regulation of β-catenin in the EGL of *β-catenin^fl/fl^* mouse, although no GFP-positive cell was detected in the region, possibly due to higher infection efficiency of AdCreM2 than Ad5-GFP in the cerebellum. Scale bars: 100 μm in (A) and (B), 20 μm in (C) and (D).
(TIF)

Figure S6 **Infection of AdCreM2 in the cerebellum.** (A and B) The pregnant dams of *R26R, β-catenin^fl/wt^* or *R26R, β-catenin^fl/fl^* mice were injected at E13.5 with AdCreM2. Sections were first stained with Xgal and then with neutral red to reveal layers. (A) Example sections from P0 mouse showing many infected cells were located in the EGL. (B) Example sections from P16 mouse showing that most Xgal-positive cells had migrated into the IGL. (C and D) The pregnant dams of *R26R, β-cat ^fl/wt^* mice or *R26R, β-cat ^fl/fl^* mice were injected at E14.5 with AdCreM2, and the offspring were analyzed at P21. (C) Example sections stained with Xgal. Some cells located in the BGL showed Bergmann glia-like morphology (arrows). (D) The sections were first stained with Xgal and then with anti-BLBP antibody using the DAB method. A few cells located in the BGL were double-positive for Xgal and BLBP (red arrows) and extended radial fibers to the pial surface. (E) A *R26R, β-cat^ fl/fl^* mouse was injected with a viral mixture of AdCreM2 plus Ad5-GFP at P0, and analyzed at P19. The section was first stained with Xgal, and then with anti-GFP antibody using the DAB method. Although Xgal-positive cells were located mostly in the IGL with no GFP expression, some were located in the BGL and positive for GFP (arrows). GFP staining showed that they extended normal radial processes to the pial surface. Left panel: staining with Xgal alone. Right panel: enlargement of the square in the middle panel. Cells showing colocalization were recognized by comparison of the left panel with the middle or right panel. Abbreviations: egl, external granule cell layer; igl, internal granule cell layer. Scale bars: 100 μm in (A), (C) and (E), 500 μm in (B), and 20 μm in (D).
(TIF)
