# Supplementary figures and images for: β-Catenin Is Critical for Cerebellar Foliation and Lamination
Source: PLoS One. 2013 May 17;8(5):e64451. doi: 10.1371/journal.pone.0064451 (PMC3656863; doi:10.1371/journal.pone.0064451)

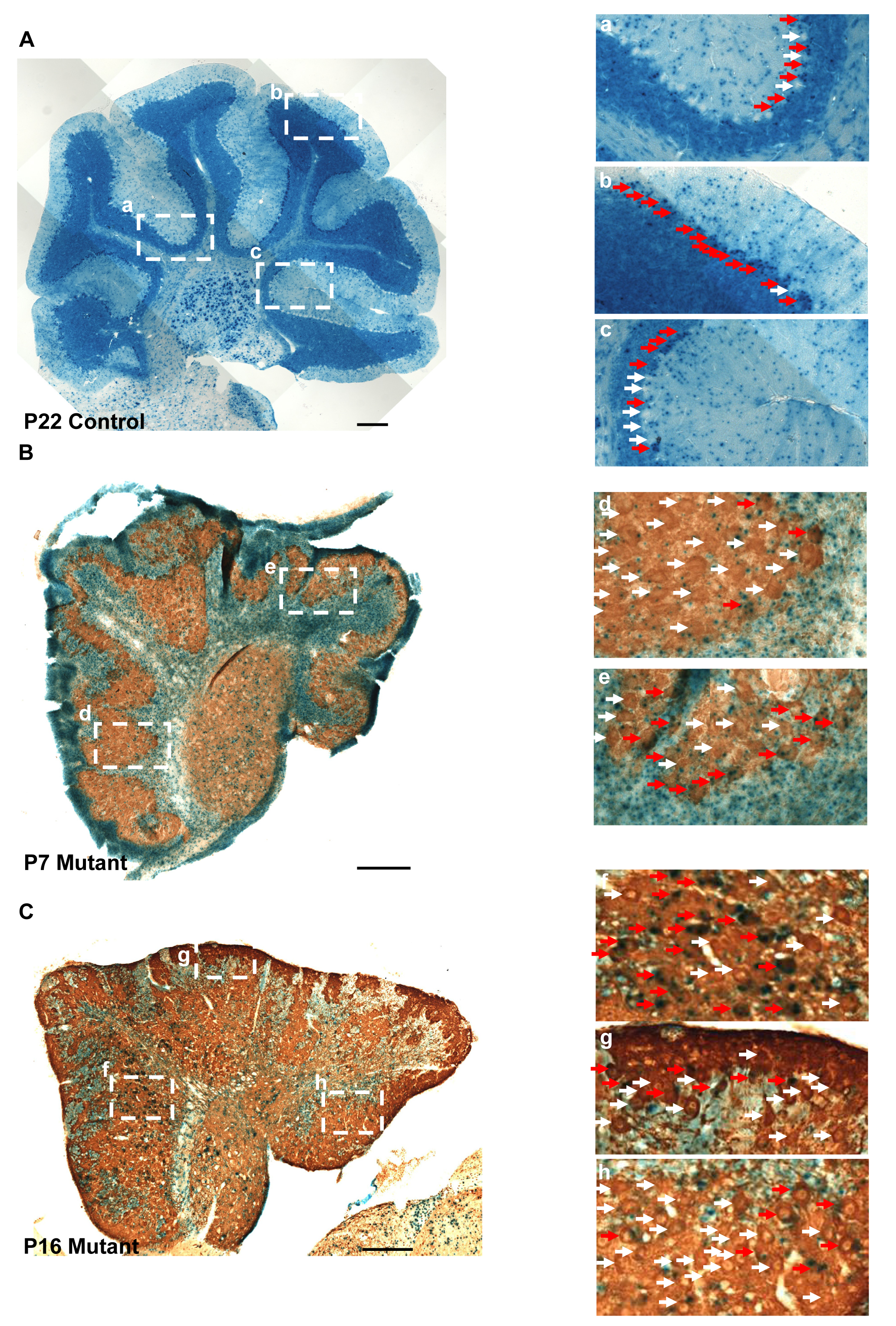

Supplement: Figure S1 — Recombination in Purkinje cells driven by the hGFAP-Cre promoter. (A) Example cerebella slice stained with Xgal from a P22 hGFAP-Cre, R26R mouse. It was the same slice shown in Fig. 1E, which was also stained with anti-Calbindin using a fluorescent secondary antibody. Only Xgal staining was shown for clarity. Right panels are enlargement of corresponding squares labeled with a-c in the left panel. (B and C) Example cerebella slices from a P7 (B) or P16 (C) hGFAP-Cre, R26R, β-cateninfl/fl mouse stained with Xgal and anti-Calbindin antibody (DAB staining). The right panels are enlargement of corresponding squares labeled with d-h in the left panels. In all the figures Xgal-positive Purkinje cells tended to form clusters (red arrows). Such clusters scattered throughout the cerebellum and mixed up with Xgal-negative Purkinje cells (white arrows). Scale bars: 250 μm. (TIF) [file pone.0064451.s002.tif]

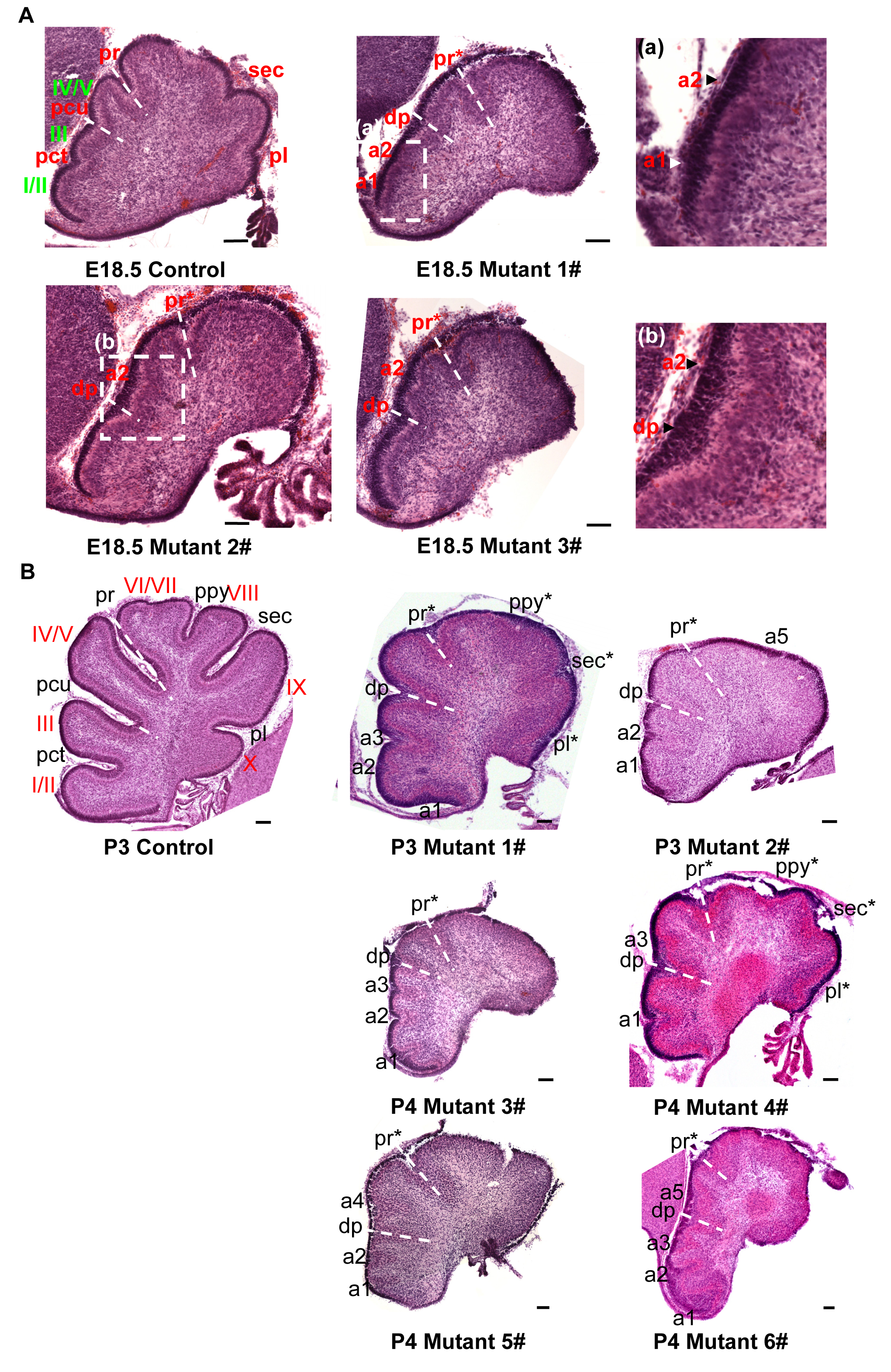

Supplement: Figure S2 — Variable foliation patterns of the hGFAP-Cre, β-cateninfl/fl mice. Midsagittal sections were analyzed by the hematoxylin and eosin method. The white dotted line indicates the position of the pr, or the deepest fissure in the anterior lobe (pcu in the control mice and dp in the mutant mice). In the mutant mice recognizable fissures were designated by an asterisk after the corresponding name. Other fissures were assigned from rostral to caudal as ‘a1, a2...an’. (A) Example sections of three E18.5 mutant mice. Mutant 1# had two fissures (a1 and a2) anterior to the dp. In the control mouse there was only one fissure (pct) anterior to the deepest fissure (pcu). Mutant 2# and 3# had one ectopic fissure between the dp and pr. The right panels are enlargement of the squared areas in the left panels (a and b). (B) Sections of six P3 or P4 mutant mice. They were different from each other. Anterior to the dp, there were two (mutant 2# and 5#) or three (mutant 1#, 3# and 6#) fissures. In the control mouse there was only one (pct). Between the pr and dp, mutant 1#, 2# and 3# had no ectopic fissure, whereas mutant 4#, 5# and 6# had one ectopic fissure (a3 in 4#; a4 in 5#; a5 in 6#). Scale bars: 100 μm. (TIF) [file pone.0064451.s003.tif]

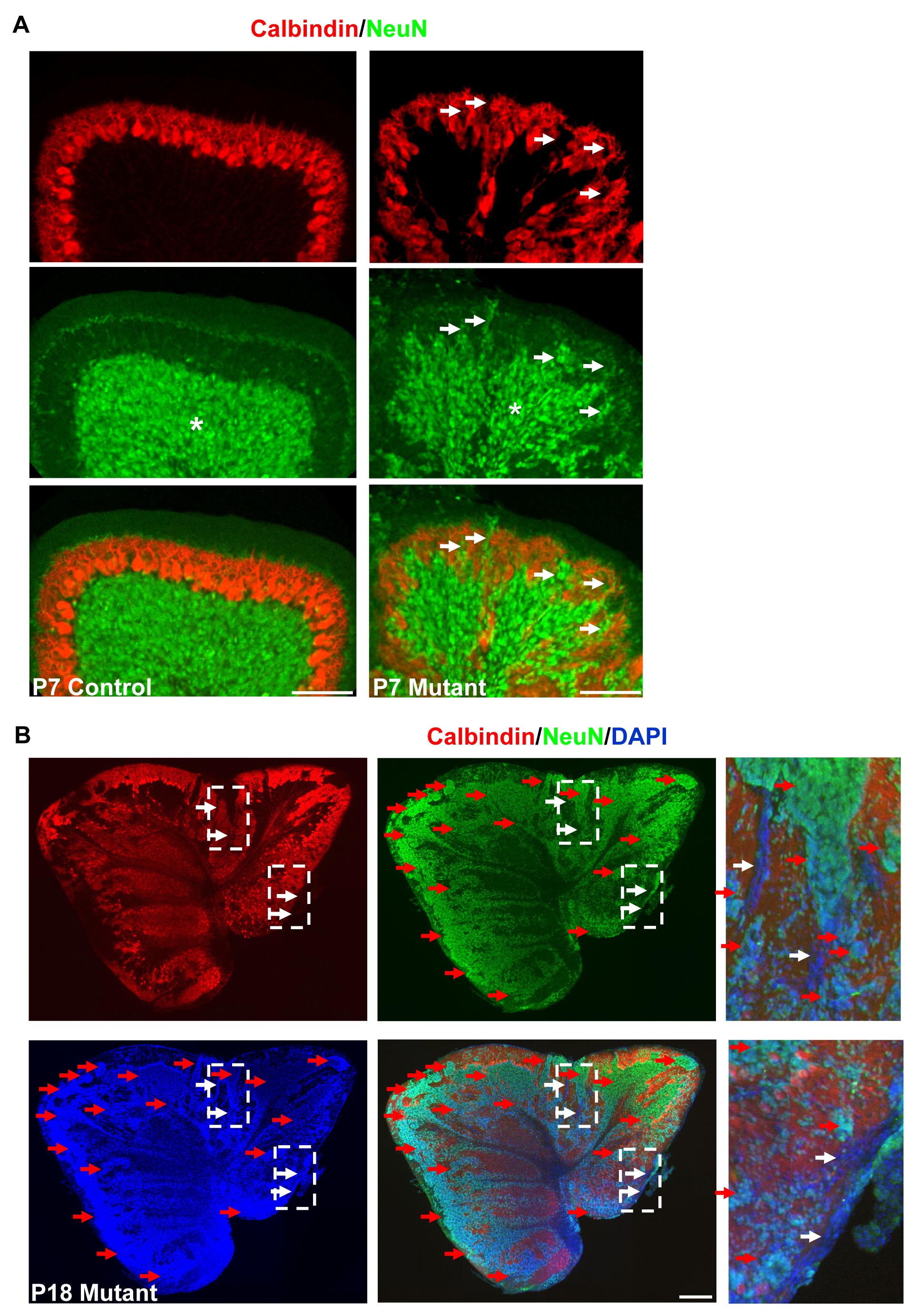

Supplement: Figure S3 — Distribution and differentiation of granule cells in the hGFAP-Cre, β-cateninfl/fl mouse. (A) Parts of the P7 control and mutant cerebellum in Fig. 5 were enlarged and shown. In the mutant cerebellum many granule cells had migrated inward and formed the IGL (asterisk), whereas some granule cells showed retention in the gaps of Purkinje cell dendrite layer (arrows). (B) The P18 mutant cerebellum was stained with DAPI and antibodies against Calbindin and NeuN. Granule cells tended to accumulate and thus were largely recognized as DAPI-positive accumulations. Note that most cells in DAPI-positive accumulations were NeuN-positive (red arrows). Only very few cell accumulations were NeuN-negative (white arrows), suggesting that most granule cells had undergone differentiation. Scale bars: 100 μm in (A) and 250 μm in (B). (TIF) [file pone.0064451.s004.tif]

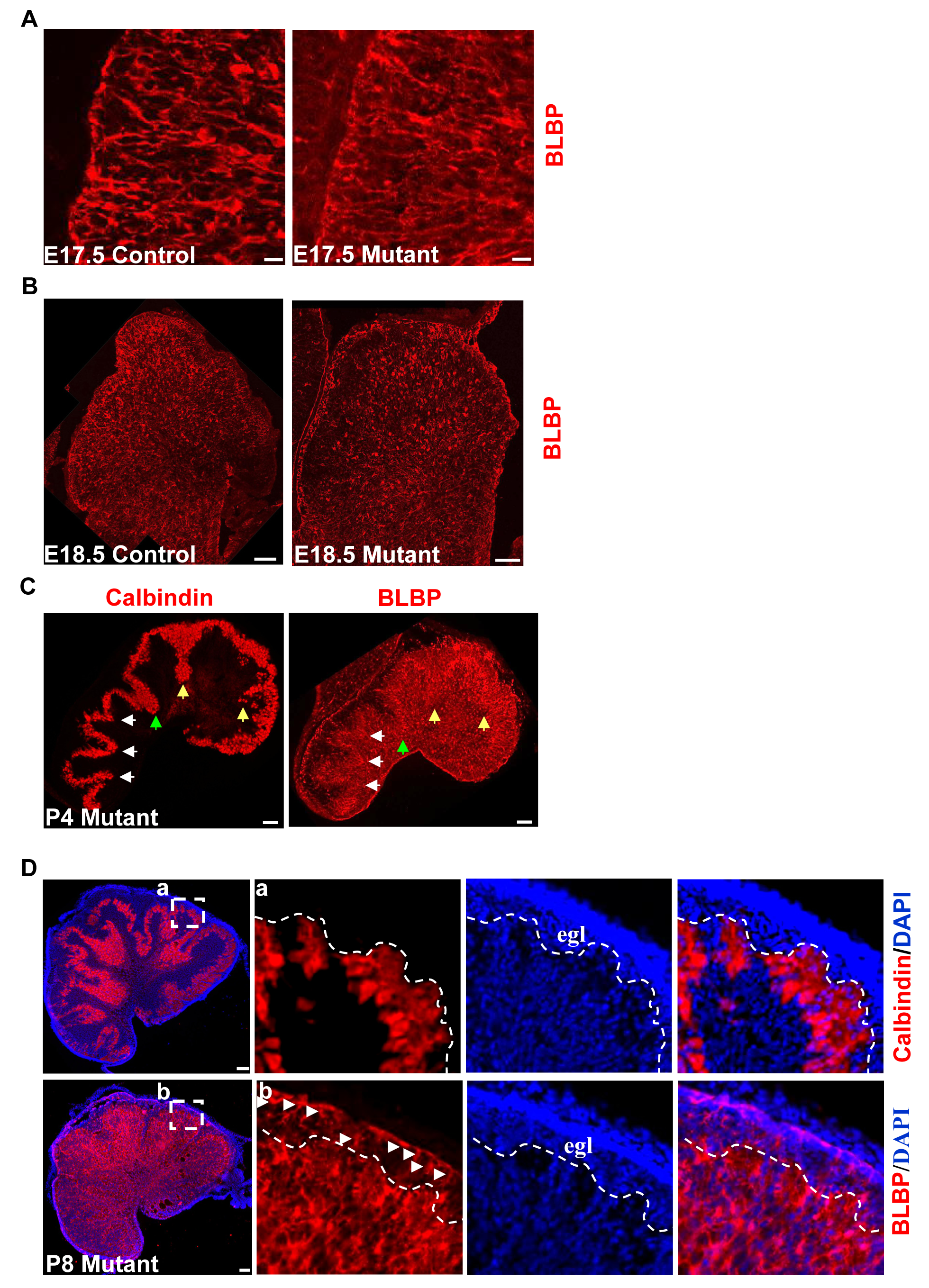

Supplement: Figure S4 — Defects in Bergmann glia of the hGFAP-Cre, β-cateninfl/fl mouse. (A) Example sections showing radial fibers spanned the EGL in the rostral part of E17.5 control and mutant cerebellum. (B) Example sections showing ectopic cell bodies appeared in the EGL of the E18.5 mutant cerebellum. (C) Neighbouring sections from a P4 mutant mouse were stained with antibodies against Calbindin or BLBP, revealing similar foliation pattern. White arrows indicate separated fissures. Yellow arrows indicate fused fissures. The green arrow indicates a fissure fused at the base. (D) Midsagittal sections from a P8 mutant mouse were stained with antibodies against Calbindin or BLBP and counterstained with DAPI. The squared region in the left panel were enlarged and shown in the three right panels. The dotted lines indicate the border of inner egl, which was corresponding to the border of dendritic terminal of Purkinje cells (a) or compact astroglia in the interior of cerebellar cortex (b). The arrowheads indicate the scattered astroglia beneath the pial surface or in the egl. Scale bars: 20 μm in (A), 100 μm in (B), (C) and (D). (TIF) [file pone.0064451.s005.tif]

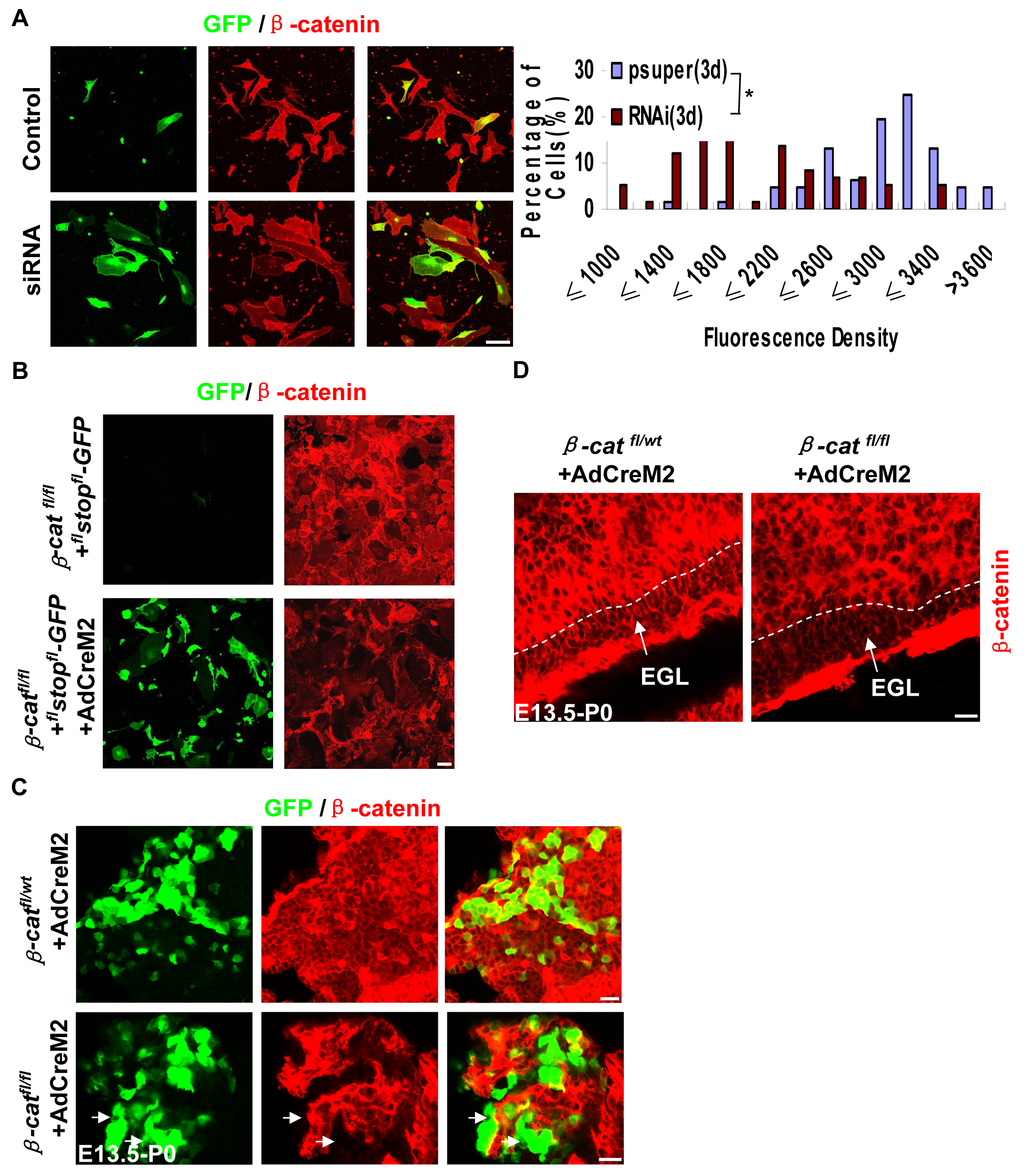

Supplement: Figure S5 — Inhibition of β-catenin expression by siRNA or Cre-expressing virus. (A) Cultured cortical astrocytes were electroporated with pSuper vectors carrying either control siRNA or β-catenin siRNA. After 3 days, cells were fixed and immunostained with anti-β-catenin antibody. The expression level (mean fluorescence density) of β-catenin within individual GFP-positive cells was quantified. The percentages of cells within each range of expression are shown in the histograms. Differences were statistically significant (p<0.001) as determined by the Mann-Whitney two-sample rank sum test. (B) Astrocytes from β-catfl/fl mouse were cultured and transfected with a plasmid containing a floxp-flanked stop sequence between a ubiquitous promoter and the EGFP gene (flstopfl-GFP). The cells were fixed and immunostained with anti-β-catenin antibody 6 days after infection with or without AdCreM2. GFP expression was initiated and β-catenin expression was inhibited in the AdCreM2-infected cells. Note that AdCreM2 affected more cells than the flstopfl-GFP plasmid, as judged by the down-regulated β-catenin expression in some GFP-negative cells. (C) E13.5 β-cateninfl/wt (upper panels) or β-cateninfl/fl (lower panels) mice injected with a viral mixture of AdCreM2 plus Ad5-GFP. The mice were killed and the choroid plexus was stained with anti-β-catenin antibody at P0. GFP-positive cells of the β-cateninfl/fl mouse showed an apparent decrease in the expression level of β-catenin (arrows). (D) The cerebella from the same mice as in (C) were stained with anti-β-catenin antibody. Note an apparent down-regulation of β-catenin in the EGL of β-cateninfl/fl mouse, although no GFP-positive cell was detected in the region, possibly due to higher infection efficiency of AdCreM2 than Ad5-GFP in the cerebellum. Scale bars: 100 μm in (A) and (B), 20 μm in (C) and (D). (TIF) [file pone.0064451.s006.tif]

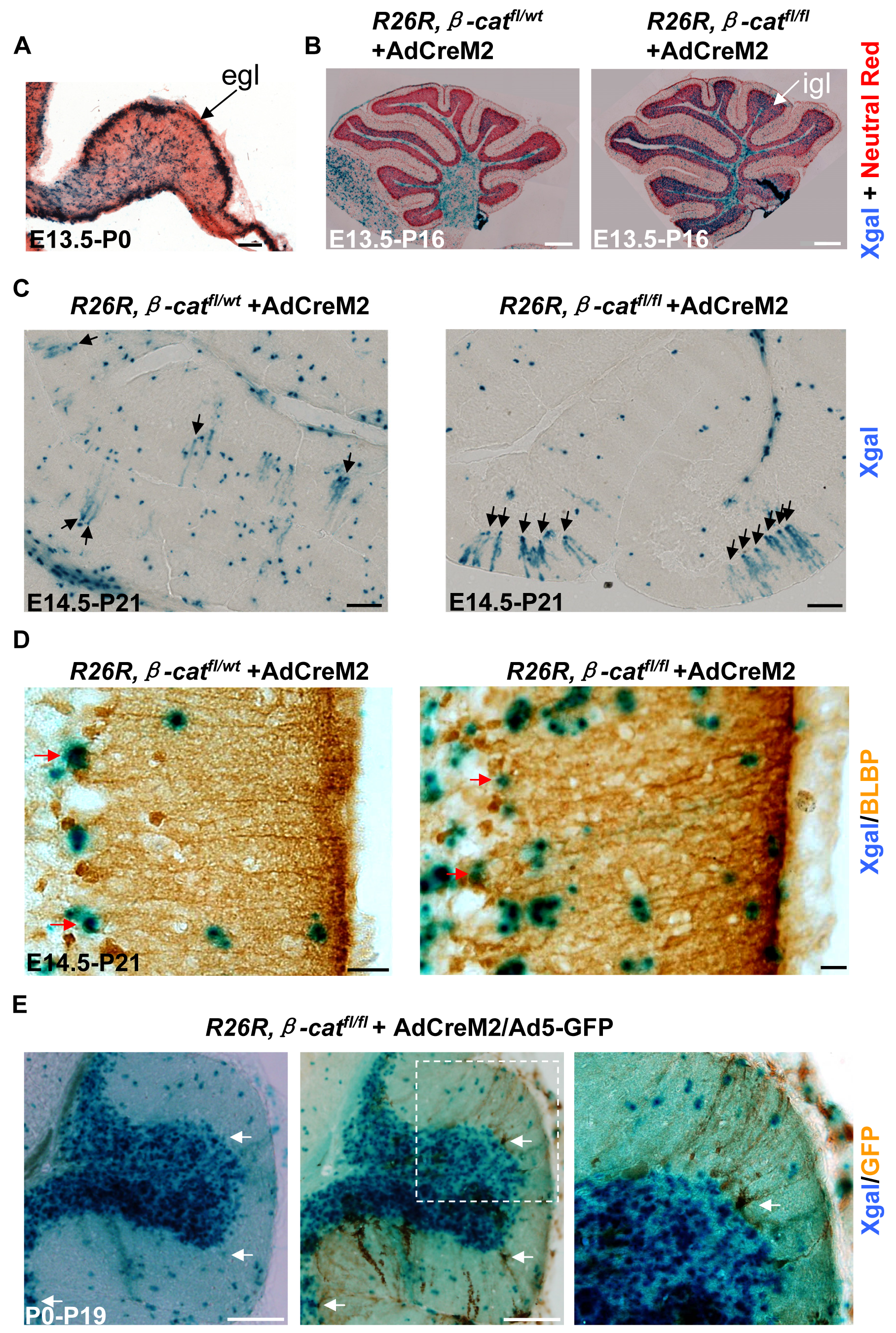

Supplement: Figure S6 — Infection of AdCreM2 in the cerebellum. (A and B) The pregnant dams of R26R, β-cateninfl/wt or R26R, β-cateninfl/fl mice were injected at E13.5 with AdCreM2. Sections were first stained with Xgal and then with neutral red to reveal layers. (A) Example sections from P0 mouse showing many infected cells were located in the EGL. (B) Example sections from P16 mouse showing that most Xgal-positive cells had migrated into the IGL. (C and D) The pregnant dams of R26R, β-cat fl/wt mice or R26R, β-cat fl/fl mice were injected at E14.5 with AdCreM2, and the offspring were analyzed at P21. (C) Example sections stained with Xgal. Some cells located in the BGL showed Bergmann glia-like morphology (arrows). (D) The sections were first stained with Xgal and then with anti-BLBP antibody using the DAB method. A few cells located in the BGL were double-positive for Xgal and BLBP (red arrows) and extended radial fibers to the pial surface. (E) A R26R, β-cat fl/fl mouse was injected with a viral mixture of AdCreM2 plus Ad5-GFP at P0, and analyzed at P19. The section was first stained with Xgal, and then with anti-GFP antibody using the DAB method. Although Xgal-positive cells were located mostly in the IGL with no GFP expression, some were located in the BGL and positive for GFP (arrows). GFP staining showed that they extended normal radial processes to the pial surface. Left panel: staining with Xgal alone. Right panel: enlargement of the square in the middle panel. Cells showing colocalization were recognized by comparison of the left panel with the middle or right panel. Abbreviations: egl, external granule cell layer; igl, internal granule cell layer. Scale bars: 100 μm in (A), (C) and (E), 500 μm in (B), and 20 μm in (D). (TIF) [file pone.0064451.s007.tif]
